# Observations in incorporating lung ultrasound views into the echo lab: value in decompensated heart failure

**DOI:** 10.1186/s12872-025-05061-4

**Published:** 2025-08-04

**Authors:** Mariam B. Camacho, Samantha R. Spierling Bagsic, Santiago Camacho, James N. Phan, Bruce J. Kimura

**Affiliations:** 1 Department of Medicine, Scripps Mercy Hospital, San Diego, CA USA; 2 Department of Research and Development, Scripps Health, San Diego, CA USA; 3 Noninvasive Cardiology, Scripps Mercy Hospital, 4077 Fifth Ave, San Diego, CA 92103 USA

**Keywords:** Congestive heart failure, Lung ultrasound, B-linesDiagnostic accuracy

## Abstract

**Background:**

Although lung ultrasound (LUS) can detect specific findings in decompensated congestive heart failure (dCHF), it is largely unavailable to hospitalized patients outside of point-of-care ultrasound practice. Therefore, we sought to determine if 4 lung views added value to echocardiography and whether LUS could be performed expeditiously in an inpatient echo lab.

**Methods:**

Consecutive inpatient echo studies from a 300-bed community hospital included two posterobasal and two anteroapical lung views and were retrospectively reviewed for: (1) echo parameters including EF < 40%, E/e’>13, pseudonormal E/A ratio, among others, of which the presence of any one parameter defined an abnormal echocardiogram, Echo+, and (2) LUS bilateral findings of 3-or-more B-lines or pleural effusion defined an abnormal lung study, LUS+. Patient charts were reviewed for the clinical diagnosis of dCHF as the reference standard. Diagnostic accuracies were determined for Echo, LUS, and their combination in predicting dCHF by univariate and area under the receiver-operating characteristic (AUC) analyses. The time necessary to perform the LUS was recorded.

**Results:**

Of *n* = 129 inpatients, mean (±SD) patient age was 67.0 ± 16.3 years, 57% were male, 32/129 (25%) had dCHF. LUS + was present in 65/129 (50%) and was related to dCHF (*p* < 0.0001). Despite the high 91% sensitivity of Echo + alone, the addition of LUS findings improved specificity from 49 to 89% and accuracy from 60 to 84%. Lung imaging views required only 95 s ± 42 [range: 30–227] to perform.

**Conclusions:**

The addition of 4 simple lung views to the standard echocardiogram improves diagnostic accuracy for decompensated CHF without increasing imaging resources. These pilot data support integrating lung ultrasound with standard echocardiography for healthcare delivery in hospital settings.

**Supplementary Information:**

The online version contains supplementary material available at 10.1186/s12872-025-05061-4.

## Background

Lung ultrasound (LUS) could be considered indispensable in cardiovascular medicine as it provides both diagnostic and prognostic data in congestive heart failure [1–4]. Ultrasound findings of B-lines and pleural effusions, indicative of extravascular lung water [5, 6] provide evidence of lung involvement in “decompensated” congestive heart failure, confirming a progression from the echocardiographic determination of requisite elevated LA pressures. In the United States and other countries, the current use of LUS in congestive heart failure (CHF) has largely been a part of point-of-care ultrasound (POCUS) [7, 8], often performed by specialty-trained physicians in emergency and critical care medicine, while CHF evaluation by standard echocardiography is provided by cardiologists through cardiac sonographers. Most practicing physicians are not familiar with LUS, largely due to a lack of exposure and access to it in mainstream diagnostic pathways. To date, it is difficult for a general physician to obtain a LUS through a department within the hospital, thereby dramatically limiting the field to a small group of trained subspecialists who must obtain equipment and perform the technique themselves.

As hospitalized patients with unexplained dyspnea are often sent for echocardiography, it is possible that the inclusion of an abbreviated LUS exam could provide diagnostic information for decompensated heart failure without a need for additional hospital resources. However, incorporating a LUS exam into every echo must be justified by (1) a significant prevalence of CHF in patients referred for echocardiography, (2) an added benefit of LUS data to identify decompensation, and (3) expediency when performed by a cardiac sonographer. As little data exist on the above assumptions, we sought to observe data from an inpatient echo laboratory practicing a blended cardiac and LUS exam in all standard echocardiograms. Specifically, we sought to observe the additional time required and diagnostic accuracy afforded by the incorporation of a simplified (4-view) lung ultrasound exam into consecutive echocardiograms, which routinely measure parameters of LA pressure.

## Methods

**Fig. 1:**
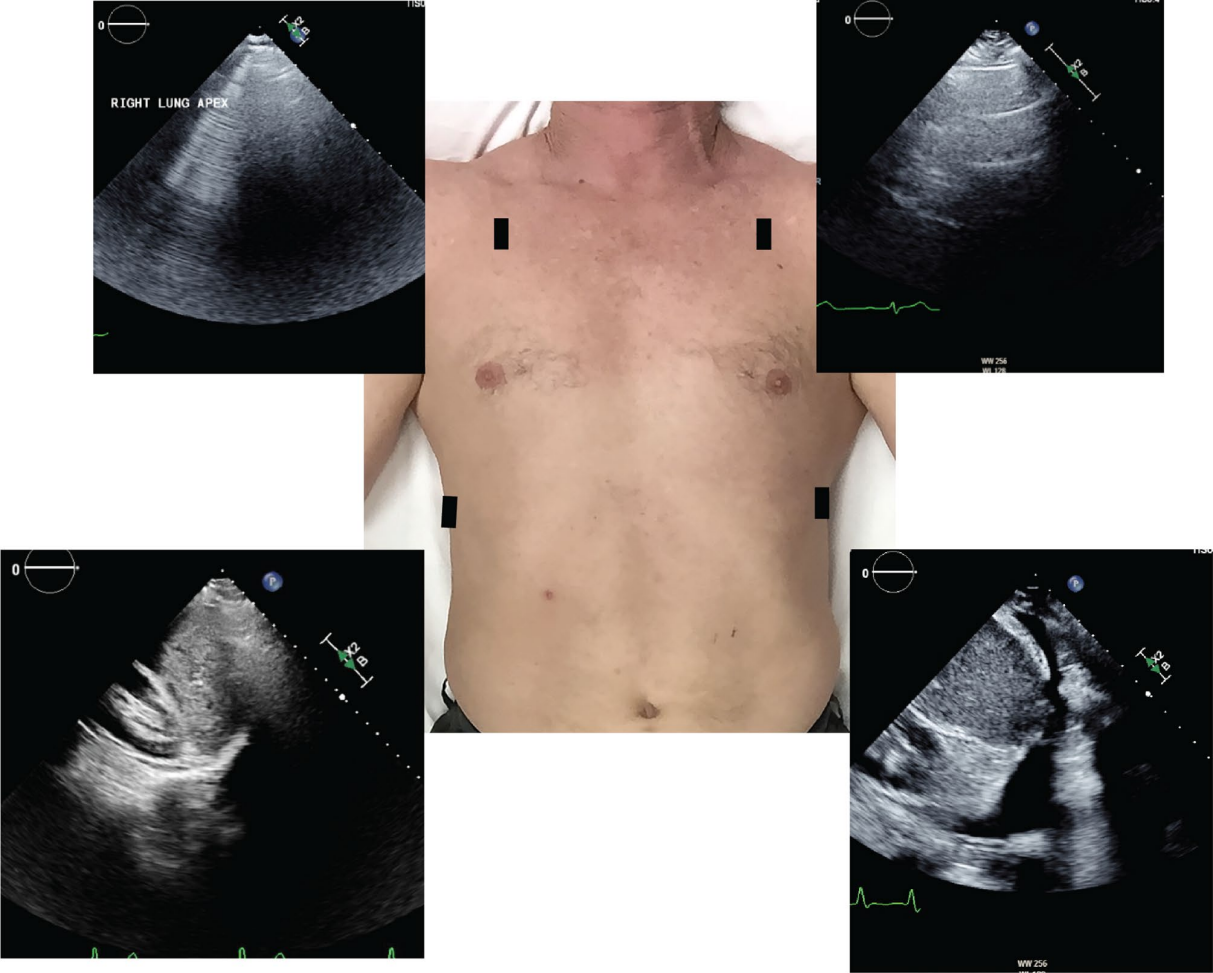
4-view lung ultrasound exam: Lung ultrasound exam included 2D imaging only in the longitudinal (parasagittal) view in the lung apex (intercostal space #3, mid-clavicular line) and posterior coronal views of the lower lobes at the diaphragm. Orientation of the image maintains the cardiac convention, cephalad and directional markers on the right of the screen, for seamless acquisition by the sonographer. Sample B-lines (top-left) and pleural effusion (bottom-right) shown

This study is a retrospective review of 129 consecutive, technically adequate adult echo studies at an inpatient echocardiography lab at Scripps Mercy Hospital in San Diego from 10/29/2022-11/07/2022, having excluded only 1 study for being technically difficult. The study was approved by the institution’s review board (ScrippsHealth IRB #22-8067). All patient data was de-identified as advised by the IRB due to the retrospective nature of this study. At this 300-bed institution, the standard echocardiographic imaging protocol has included four lung views, based upon clinical observations and outcomes over the past decade [4, 9, 10]. All imaging was done using a 3 MHz transducer (Epic, Philips Healthcare) by a certified sonographer with the patient in supine position for the duration of the entire examination. All 18 sonographers at Scripps Mercy Hospital have been registered in cardiac imaging and were also trained within the lab to incorporate a 4-view lung ultrasound as part of the standard echocardiogram. The LUS training has included hands-on demonstrations and critiques of subsequent applications in lab quality assurance meetings, similar to the ongoing teaching of new echocardiographic techniques, and sonographers typically become proficient within 1 week (10–20 studies) of practice and proctoring by senior sonographers. The 4 lung views (Fig. 1) are routinely performed at the end of every echocardiogram using the cardiac preset (mechanical index = 1.3, depth 16 cm, proximal focus) with the patient in supine position. All echo images were reviewed and interpreted by the echo panel reader-of-the-day, a board-certified cardiologist. For this study’s data collection, lung images were independently re-interpreted by a separate cardiologist who is an expert in the lung ultrasound field and blinded to patient and echo data. To assess the time needed to perform the lung imaging, time stamps were used to determine the durations of individual components of the entire exam.

An abnormal echo in this study, in regards to congestive heart failure, Echo(+), was defined by the presence of any one or more of the following: (1) ejection fraction (EF) *≤* 40%, (2) inferior vena cava (IVC) plethora where the IVC was dilated greater than the neighboring aorta and did not show 50% diameter reduction with inspiration [11], (3) the presence of at least moderate-to-severe valvular stenosis or regurgitation, (4) left atrial volume index (LAVI) *≥* 34 ml/m^2^, (5) E/A > 1.2 if age *≥* 65 (“pseudonormal” pattern) as inferred from population data [12, 13], or (6) E/e’ (using lateral wall e’) of > 13 [14, 15]. Atrial fibrillation confounds the use of E/A and E/e’ parameters and those values were not included in the analysis of Echo(+). A subgroup analysis was performed in patients with atrial fibrillation.

The lung imaging was conducted by the same sonographer who completed the standard cardiac echocardiogram described above. While extensive lung imaging protocols have previously been described that utilize up to 28 views, the 4-view lung imaging protocol used in this study had been routinely employed by the lab based upon prior evidence of the prognostic value of 3-or-more B-lines in the lung apices and pleural effusions in the bases [4, 9, 10], the relative ease in which it can be taught to cardiac sonographers, and its expedient integration into the echo study. An abnormal lung ultrasound in this study, LUS(+), was defined by the presence of any one or more of the following when observed bilaterally: (1) 3-or-more B-lines in the lung apex (second or third intercostal space in the midclavicular line) and/or (2) pleural effusions or 3-or-more B-lines in the posterolateral lung base [4–6, 9, 10].

As there is no universal, agreed-upon definition of clinical decompensation in the congestive heart failure literature, the study constructed a definition of dCHF by combining clinical, serologic, and radiographic findings. Patient charts were reviewed for the clinical diagnosis of dCHF which was defined as the presence of at least two of the following: (1) elevated NT-proBNP (*≥* 1800, regardless of age), (2) discharge diagnosis of CHF exacerbation (or related discharge diagnosis such as HFrEF, heart failure with reduced ejection fraction, or HFpEF, heart failure with preserved ejection fraction) with assessment and plan documentation of active inpatient interventions as an active problem, and (3) compatible radiographic findings as observed on X-Ray (XR) or computerized tomography (CT) scan, by the radiologist, including mentioning of terms such as CHF, pleural effusion, interstitial or pulmonary edema in the formal report. As the discharging physician was not blinded to the echo report when determining the overall discharge diagnosis, the study required at least 2 of the 3 criteria to be present in the definition of decompensated CHF.

Statistical analyses were conducted by evaluating the diagnostic accuracies, sensitivities, specificities, and area under receiver-operating curves (AUC) for echo and LUS findings relative to clinical diagnosis of dCHF. There was no guidance from prior literature to reliably power this retrospective, observational study a priori. We aimed for this preliminary study to provide pilot data for future, adequately powered trials. A previous outcome study from this laboratory reported a 40% prevalence of an abnormal LUS in consecutive inpatient referrals [4], and based upon hospital experience, we assumed at least half of all referrals would be due for evaluation of CHF. Therefore, we sought at least one hundred consecutive hospitalized patients in whom to observe imaging time and diagnostic accuracy to balance feasibility and efficiency in collecting this important preliminary data. Univariate associations were tested using Fisher’s Exact test. Confidence intervals for AUC for each imaging modality or combination of modalities are reported. Diagnostic statistics were computed using the pROC package in R. All statistical analyses were conducted in R v. 4.1.3 in the RStudio environment.

## Results

Of *N* = 129 consecutive hospitalized patients, the mean (±SD) patient age was 67.0 ± 16.3 years, 57% were male, 32/129 (25%) had dCHF by chart review (Table 1). Admitting diagnoses were variable, but included dyspnea 17/129 (13%), sepsis or septic shock 11/129 (9%), stroke 12/129 (9%), syncope 8/129 (6%), among others. 53/129 (41%) identified as never smokers, 18/129 (14%) as current smokers, and the smoking status of the remaining subjects was unclear from chart review.

LUS + was present in 65/129 (50%) and was associated with dCHF (p < 0.0001). Nearly all the individual echo parameters demonstrated a significant (p *≤* 0.05) relationship with dCHF, except E/e’ and E/A, neither of which was found to be statistically significant (Table 1). Echo(+), when defined as any one of the echo parameters as being present, demonstrated a sensitivity = 0.91 for detecting dCHF; however, much less in regards to specificity (0.49), with an overall accuracy of 0.60 (Table 2, line 1). The combination of standard echocardiography and lung ultrasound yielded higher accuracy and specificity (accuracy = 0.84, specificity = 0.89, sensitivity = 0.69) for detecting dCHF than either modality by itself (Table 2, line 3). Using time stamp differences, the addition of lung ultrasound to the standard echocardiogram required 95 s ± 0:42 [range:30–227], an additional 7% of imaging time overall on average.

Implementation of these results in clinical practice could resemble a stepwise approach and vary based on the needs of the institution. Table 2 demonstrates a model whereby LUS is conducted in response to Echo(+), contributing to the specificity and accuracy of the study whilst saving time by incorporating lung views only to studies that portend potential decompensation. Atrial fibrillation was noted in 16/129 subjects (see Supplemental table) and was associated with dCHF [50% (8/16) vs. 21% (24/113), (*p* = 0.03)]. All patients with atrial fibrillation who met criteria for dCHF (8/8) were LUS+.

**Table 1:** Demographic, echocardiographic, and LUS parameters and their association with dCHF

	Overall (*n* = 129)		dCHF(-) (*n* = 97)		dCHF(+) (*n* = 32)		Significance (Fisher’s exact)
	*n*	%	*n*	%	*n*	%	*p*-value
Age > 65y	82	64%	57	59%	25	78%	0.06
Male	73	57%	58	60%	15	47%	0.22
EF ≤ 40%	16	12%	6	6%	10	31%	0.0007
IVC plethora	32	25%	17	18%	15	47%	0.002
Valvular disease	22	17%	10	10%	12	38%	0.0009
LAVI ≥ 34	43	33%	26	27%	17	53%	0.0091
E/A	26	20%	19	20%	7	22%	0.8019
E/e’	19	15%	12	12%	7	22%	0.2483
B-lines in the lung apices (bilateral)	32	25%	15	15%	17	53%	0.0001
LUS + in the lung bases (bilateral)	16	12%	4	4%	12	38%	0.0001
LUS+ (cumulative)	40	31%	17	18%	23	72%	0.0001

Table depicts the frequency of each parameter (rows) in the overall population, dCHF(-) subjects versus dCHF(+) subjects, respectively (columns). P-values displayed for univariate associations using Fisher’s exact test.* EF* ejection fraction;* IVC* inferior vena cava; Valvular disease of moderate-severe severity;* LAVI* left atrial volume index (ml/m2),* E/A* ratio of transmitral E(early) to A(atrial) peak velocities;* E/e’* ratio of transmitral E wave velocity (cm/s) to doppler tissue velocity of the lateral mitral annulus,* e’ (cm/s)* LUS + lung ultrasound abnormal findings

.

**Table 2:** Diagnostic accuracy of Echo(+), LUS(+) combination for dCHF

	Imaging modality	Sensitivity	Specificity	Accuracy	AUC (95% CI)
1	Echo+	0.91 (0.75, 0.98)	0.49 (0.39, 0.60)	0.60 (0.51, 0.68)	0.70 (0.63–0.77)
2	LUS+	0.72 (0.53, 0.86)	0.81 (0.72, 0.89)	0.79 (0.71, 0.86)	0.77 (0.70–0.87)
3	ECHO + and LUS+	0.69 (0.50, 0.84)	0.89 (0.81, 0.94)	0.84 (0.76, 0.90)	0.79 (0.58–0.77)
4	ECHO + then LUS+	0.76 (0.56, 0.90)	0.78 (0.63, 0.88)	0.77 (0.66, 0.86)	0.77 (0.67–0.87)

Table demonstrates the sensitivities, specificities, accuracies, area-under-curve, AUC, with respective 95% confidence intervals (CI). The combination of standard echocardiography and lung ultrasound, either together or in a stepwise approach, had higher accuracy and specificity for detecting dCHF than either modality by itself

## Discussion

This study observed that the incorporation of a four-view lung ultrasound performed on consecutive hospitalized patients referred for echocardiography is feasible and may have value specifically for decompensated CHF. In this pilot study in a community hospital echo lab, the substantial 25% prevalence of a clinical diagnosis of dCHF, the capability of the sonographers, and the efficiency in resource utilization of the hospital’s echo equipment may justify the inclusion of lung ultrasound into the echo imaging protocol where it increases the diagnostic specificity for decompensated CHF. The current study is unique in confirming a feasible and economical pathway in which to provide a valuable ultrasound service.

The growth of echocardiography has resulted in an ever-expanding imaging protocol, based upon continued advances and discoveries in the field. Multiple society and expert consensus statements [16–18], and textbooks [19, 20] have attempted to define a standard protocol, but rarely address the additional resources required to justify the application of the newer techniques in every echocardiogram. In the sonographer model used in the United States, the time needed to perform a standard study will relate to its cost. This imaging time is related to the complexity of the exam and the skill of the sonographer, and has been measured at 26 min per exam in data that is over 20 years old [21]. The concept of a “limited” cardiac ultrasound study, a brief exam designed for a simple indication, was developed to reduce imaging time, improve referral, and mitigate the inefficiency of obtaining unnecessary views [22–24]. However, limiting imaging time does not address the time necessary for equipment transport, patient preparation, and report generation that is required in formal referral hospital testing, all of which significantly reduce the value of performing a “limited” study in a sonographer model. Importantly, compared to other complex techniques routinely performed in today’s echo lab, the current study shows a modest imaging time of < 2 min to perform a 4-view LUS at the end of the echo study, using the same equipment and report. In POCUS practice, the imaging time could be much less, as the data are obtained to the satisfaction of the operator who is imaging the patient, as opposed to by a sonographer, intent on providing the highest quality image for a physician reader. The additional training needs of the cardiac sonographer have been minimal, as the simplicity of learning LUS has been shown previously in studies demonstrating proficiency in more extensive 10–12 view imaging protocols after only 1 day of training or 25 exams in physicians [25, 26] and after as little as 10 exams in respiratory therapists [27]. Previous studies have described even more extensive lung ultrasound protocols [28–30], such as 28-view approaches for better diagnostic accuracy, which may be a barrier to efficient implementation when applied to all referrals in a busy echo lab. Certainly, more time-intensive, quantitative approaches in lung imaging could be applied to smaller, select populations when indicated as an add-on procedure after standard echo imaging, similar to the use of echo contrast, strain, or 3D imaging in echo practice.

Diagnosing heart failure has been a longstanding interest in the echocardiography community. This study found that echocardiographic data alone are sensitive but limited in distinguishing compensated from decompensated CHF and may benefit from LUS. Although previous studies have related parameters of CHF to echocardiographic and lung ultrasound [31–33], the current investigation is unique in reporting the test characteristics of lung ultrasound using a clinical definition of “decompensated” CHF determined by chart review. The detection specifically of decompensated CHF has particular value, as it is well known that escalating symptomatology is prognostic in CHF [34], and that differentiation from other etiologies of dyspnea in that patient group, such as pulmonary embolism, COPD or pneumonia, can be critical. Echocardiographic parameters have shown variable accuracies, often measured in specific, well-controlled lab conditions with populations undergoing catheterization [32, 33]. Importantly, the singular presence of elevated left atrial pressures does not equate to pulmonary edema or “decompensation” due to variabilities in compensatory pulmonary venous and lymphatic drainage mechanisms [35, 36]. In this study, the addition of lung imaging appears to have an important complementary role for identifying decompensation, especially in a patient who manifests baseline vulnerable structural disease such as left enlargement or IVC plethora. Furthermore, the current study suggests value to lung ultrasound in patients with atrial fibrillation, wherein diastolic Doppler parameters, including E/A and E/e’, and the presence of left atrial enlargement are limited in the assessment of LA pressure.

A standardized lung ultrasound exam can be a difficult test to obtain in the hospital at the current time, as it is primarily performed as a point-of-care exam by a limited number of trained physicians. The role of lung ultrasonography in detecting pulmonary congestion and clinical improvement in dCHF is rapidly evolving. Monitoring B-line resolution has been associated with clinical improvement in hospitalized [37] and recently discharged [38] patients with dCHF and in ambulatory patients at risk for decompensation [39]. The presence of B-lines in outpatients with chronic heart failure is associated with adverse prognosis, and not correlated with EF [40]. Similar to rales in the lung base, B-lines in the bases may not be as specific for decompensation, as they may represent previous infections or gravity-dependency [5, 41]. In addition, the recommendations for transducer frequency, depth, and power settings on B-lines are only now being standardized. However, the cardiac transducer and settings have been used for decades in LUS [3, 4, 42, 43], and data acquired have shown diagnostic and outcome validation [2, 4, 9, 10, 38, 39]. Lung ultrasound using cardiac transducers at the time of echocardiography [4, 44] can also be used to detect pleural effusions, highly prevalent, 91%, in patients with decompensated heart failure [44] and related to a 33% one-year mortality in consecutive inpatients referred for echocardiography [4]. Conversely, the detection of B-lines using lung ultrasound without echocardiographic data, creates a common clinical conundrum, where noncardiogenic pulmonary edema, pulmonary fibrosis, or interstitial inflammation often confound the diagnosis of decompensated CHF and could potentially result in erroneous diuretic, steroid, and/or antibiotic therapies [45, 46]. Therefore, the novel practice of simultaneous acquisition of both cardiac and lung ultrasound data in the echo laboratory can (1) have diagnostic benefit above and beyond when the techniques are performed separately and at different times, (2) create institutional quality oversight within one department, and (3) make lung ultrasound data available to patients whose physicians do not practice point-of-care ultrasound. As the LUS protocol used does not require newer processing techniques such as harmonics, 3D, or tissue Doppler imaging, the use of standard, or even older, echo equipment increases the generalizability and scalability of this combined exam to include resource-limited settings. The cost-effectiveness is inherent in a blended exam that provides valuable clinical data without requiring more resources. Ultimately, more familiarity with LUS findings, when provided during echocardiography, could potentially drive LUS utilization, standardization, and research in the future.

The current study has limitations. This study was a sampling of a limited 2-week time at a cardiovascular ultrasound laboratory that has already integrated LUS into the echocardiogram and did not have the power to be a comprehensive analysis of diastology, filling pressures, and lung edema. Using a retrospective design, the study likely underestimated the perceived benefit of LUS, as the ordering physicians were not interviewed for their expectations of the test, particularly in helping differentiate unexplained dyspnea, where a negative LUS would be useful. The time delay between the ordering and performance of the LUS may have resulted in the resolution of findings with successful treatment in dCHF and reduced the apparent LUS sensitivity. Although the study was not intended to define the learning curve of LUS, multiple cardiac sonographers have been trained in our echo lab over the years, due to the simplicity and efficiency of the described protocol and their familiarity with diagnostic ultrasound equipment. These advantages likely accelerated their proficiency, requiring an even shorter time than the minimal requirement demonstrated in prior studies [25–27]. In the future, the simplified imaging of evidence-based targets, such as infiltrates, atelectasis, and pleural sliding, along with methods to estimate pleural effusion volume could be taught to cardiac sonographers. The clinicians caring for the patients in the study were not blinded to the echo, CT, or lab reports, which all likely influenced the “discharge diagnosis” documented in the discharge summary. However, the LUS and Doppler data were displayed equally in the body of the echo report, and the designation of dCHF required at least two of the three criteria including discharge diagnosis, NT-proBNP, and radiographic criteria, mitigating undue reliance on any one of these parameters (Methods).

## Conclusions

The current study demonstrates that lung ultrasound would complement echocardiography by increasing specificity for decompensated CHF and require minimal time and no additional resources when provided by the echo lab.

## Supplementary Information

Supplementary Material 1

## Data Availability

The data that support the findings of this study are available from the corresponding author, BJK, upon reasonable request.

## Abbreviations

LUS: Lung ultrasound
CHF: Congestive heart failure
dCHF: Decompensated CHF
EF: Ejection fraction
AUC: Area under the curve
PCWP: Pulmonary capillary wedge pressure
HFrEF: Heart failure with reduced ejection fraction
HFpEF: Heart failure with preserved ejection fraction
NTpro-BNP: N-terminal prohormone of brain natriuretic peptide
