# Supplementary material for: Observations in incorporating lung ultrasound views into the echo lab: value in decompensated heart failure
Source: BMC Cardiovasc Disord. 2025 Aug 4;25:579. doi: 10.1186/s12872-025-05061-4 (PMC12323090; doi:10.1186/s12872-025-05061-4)
Supplement: Supplementary file 1 — Supplementary Material 1 [file 12872_2025_5061_MOESM1_ESM.docx]

**Supplemental table:** Presence of atrial fibrillation and corresponding dCHF (decompensated CHF) status

|  | **dCHF(+)** | **dCHF(-)** | **Total** |
| --- | --- | --- | --- |
| **Atrial fibrillation (+)** | 8 | 8 | 16 |
| **Atrial fibrillation (-)** | 24 | 89 | 113 |
| **Total** | 32 | 97 | 129 |
